# Accuracy and precision guidelines for optimal breeding time in bitches using in-house progesterone measurement compared with chemiluminescent microparticle immunoassay

**DOI:** 10.14202/vetworld.2021.585-588

**Published:** 2021-03-08

**Authors:** Nutnaree Kunanusont, Darsaniya Punyadarsaniya, Sakchai Ruenphet

**Affiliations:** 1 Clinic for Horse, Faculty of Veterinary Medicine, Mahanakorn University of Technology, Bangkok, Thailand; 2 Department of Immunology and Virology, Veterinary Medicine Faculty, Mahanakorn University of Technology, Bangkok, Thailand

**Keywords:** bitches, chemiluminescent microparticle immunoassay, optimal breed timing, progesterone, rapid fluorescence immunochromatography assay

## Abstract

**Background and Aim:**

The concentration of serum progesterone is commonly used to determine the optimal mating time in bitches, and to diagnose reproductive-related abnormalities. This study aims to compare the serum progesterone results obtained by rapid fluorescence immunochromatography assay (RFICA) with those obtained by chemiluminescent microparticle immunoassay (CMIA) from the same serum samples to develop a standard guideline for optimal breeding time.

**Materials and Methods:**

Serum progesterone levels were measured in 124 bitches using RFICA and CMIA. Simple linear regression and correlation analyses were performed to analyze the data. The percentage difference between the maximum and minimum progesterone values in the same serum sample in the same assay was compared using Wilcoxon’s rank-sum test.

**Results:**

The present study showed a strong linear dependence of the results obtained by RFICA on those obtained by CMIA as R^2^=0.8976, with regression coefficient of 0.9474 and p<0.05, including the regression model was CMIA = (0.9483 × RFICA) − 0.761. Moreover, five critical measurement times during estrous in bitches showed statistically significant differences (p<0.05), except at the fertilizable period, which showed a non-significant difference (p>0.05).

**Conclusion:**

This study demonstrated that it is presumably acceptable to use the RFICA and CMIA methods interchangeably for quality progesterone measurements in serum samples from bitches. However, when considering the use of the RFICA method, it is advisable to carefully interpret the results and follow the interpretation guidelines. Finally, RFICA in the present study provides a reliable and convenient option for veterinarian practitioners to measure canine progesterone levels in-house.

## Introduction

The concentration of serum progesterone is commonly used to determine the optimal mating time in bitches [1-3], including the assessment of reproductive abnormalities such as ovarian dysfunction during pregnancy (e.g., hypoluteoidism) [4,5] and estrous cycle manipulation, especially verification of luteolysis before parturition [6]. Edens [7] and Olson *et al*. [8] reported serum progesterone concentrations of the estrous cycle as (1) above basal concentration at >1 ng/mL, indicating proestrus stage; (2) 2.0 ng/mL indicating the day of the luteinizing hormone (LH) surge; (3) 4-10 ng/mL on the ovulation day; and (4) >5 ng/mL indicating post-ovulation.

Several qualitative, quantitative, and semi-quantitative methods for progesterone measurements are available for veterinarian practitioners. In general, quantitative assays are preferred, such as radioimmunoassay (RIA) [9,10], liquid chromatography-tandem mass spectrometry [11-13], the more recently introduced enzyme-linked fluorescence assay, and chemiluminescence immunoassay (CLIA) [10,14-16].

However, serum progesterone measurement results show variations due to different laboratory methods and bitches. Therefore, accurate determination of optimal breeding time requires collection of several serial blood samples during proestrus and estrous stages to compare against the gold standard or reference laboratory methods. Thus, in this study, serum progesterone results obtained by rapid fluorescence immunochromatography assay (RFICA) were compared with those obtained by chemiluminescent microparticle immunoassay (CMIA) for the same serum samples to determine a standard guideline for optimal breeding time.

## Materials and Methods

### Ethical approval and Informed consent

Guidelines used for the care and use of animals were approved by the Animal Research Ethics Committee, Faculty of Veterinary Medicine, Mahanakorn University of Technology, Thailand, approval number ACUC-MUT-2020/006. A consent form was signed by the bitch owners to participate in the study.

### Study period and location

Blood samples were collected from August 2019 to July 2020 at the Small Animal Teaching Hospital, Faculty of Veterinary Medicine, Mahanakorn University of Technology, Thailand, and Vet Home Polyclinic, Bangkok, Thailand.

### Sample collection

One-hundred and twenty-four bitches of different breeds such as the American bullies, English bulldog, French bulldog, Chihuahuas, Pomeranian, Chow Chow, Akita, and Pug were hospitalized for routine estrous observation and artificial insemination at the Small Animal Teaching Hospital, Faculty of Veterinary Medicine, Mahanakorn University of Technology, Thailand, and Vet Home Polyclinic, Bangkok, Thailand. Blood samples were collected from all the bitches by performing venipuncture 5-7 days after the onset of vaginal swelling or discharge. Blood was allowed to clot and then centrifuged at 3000 g for 7 min. The harvested sera were transferred into microtubes and stored at 30°C until required for testing.

### Progesterone measurements

The concentration of progesterone in each serum sample was determined by (1) RFICA using a Dianotech Fluorescence Quantitative Analyzer and canine progesterone rapid test kit (Beijing Dianotech Science and Technology Co., Ltd., Beijing, China) and (2) by CMIA using an Architect i2000SR Immunoassay Analyzer and Reagent Architect Progesterone (Abbott Laboratories, Illinois, USA).

### Statistical analysis

Statistical methods involved simple linear regression and correlation analyses to determine the relationship between the RFICA and CMI. A correlation coefficient of ≤0.35 was defined as low or weak correlation, 0.36-0.67 as moderate correlation, 0.68-0.89 as high correlation, and >0.90 as very high correlation [17]. The square root of RFICA was regressed on the square root of CMIA. Squaring both sides of the derived regression equation provided a formula for predicting RFICA from CMIA. Data analysis was performed using Stata 14 (Stata Corp, College Station, TX, USA) and Excel.

Percentage differences between maximum and minimum progesterone values on the same serum sample in the same assay were compared using the Wilcoxon’s rank-sum test.

## Results

**Figure-1:**
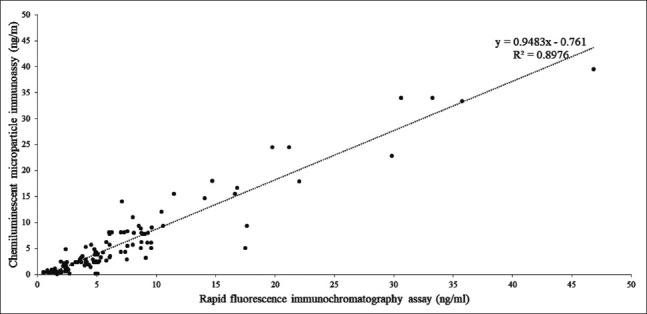
Correlation graph of progesterone measurement between rapid fluorescence immunochromatography assays compared with chemiluminescent microparticle immunoassay.

Figure-1 shows a strong linear dependence of the results obtained by RFICA on those obtained by CMIA as R^2^=0.8976, with regression coefficient of 0.9474 and p<0.05. The regression model was CMIA = (0.9483×RFICA)−0.761.

The ratio of the standard deviation to the mean, namely, the coefficient of variation, for critical times during estrus in bitches such as stages of anestrus; namely, proestrus or pre-LH surge, LH surge, post-LH surge and pre-ovulation, at or near ovulation day, and fertilizable period, was 71.12%, 8.54%, 7.24%, 38.89%, and 25.56% for CMIA and 38.46%, 8.46%, 6.93%, 28.18%, and 46.87% for RFICA, respectively.

The results of progesterone levels obtained using RFICA were higher than those obtained using CMIA. On the day that CMIA showed stages of anestrus, proestrus or pre-LH surge, RFICA measured higher than CMIA in 44 of 44 bitches sera (100%, p<0.001), similar to the stage of LH surge (19 of 20; 95%, p<0.001), post-LH surge and pre-ovulation (8 of 8; 100%, p=0.016), and at or near ovulation day (22 of 36; 61.11%, p=0.026). However, during the fertilizable period, the progesterone level shown by RFICA was lower than that shown by CMIA in 10 of 16 bitch sera (62.50%, p=0.565) (Table-1).

**Table-1:** Concentrations of progesterone measured with RFICA and CMIA and critical times during estrus in bitches.

Stage of estrous cycle	Minimum	25^th^ percentile	Median	75^th^ percentile	Maximum	Mean	SD	n	p
Anestrus, proestrus, and pre-LH surge (below 2.00 ng/mL)									
CMIA	0.1	0.40	0.58	1.37	1.97	0.83	0.58	44	<0.001
RFICA	0.5	1.28	1.97	2.46	5.04	2.14	1.17	44	
LH surge (2.00-2.99 ng/mL)									
CMIA	2.21	2.35	2.40	2.66	2.89	2.51	0.21	20	<0.001
RFICA	1.95	3.43	4.07	4.91	7.51	4.16	1.25	20	
Post-LH surge and pre-ovulation (3.00-3.99 ng/mL)									
CMIA	3.16	3.26	3.45	3.75	3.82	3.47	0.24	8	0.016
RFICA	3.66	4.05	5.20	6.09	9.13	5.49	1.62	8	
At or near ovulation (4.00-10.00 ng/mL)									
CMIA	4.2	5.07	6.21	8.07	9.40	6.70	1.69	36	0.026
RFICA	2.37	6.03	7.58	9.01	17.64	7.83	3.00	36	
In fertilizable period (10.01-40.00 ng/mL)									
CMIA	11.05	14.88	18.00	31.15	39.52	21.77	8.73	16	0.565*
RFICA	7.09	12.15	18.28	30.44	46.81	21.16	10.88	16	

* Non-significant difference (p>0.05). RFICA=Rapid fluorescence immunochromatography assay, CMIA=Chemiluminescent microparticle immunoassay, LH=Luteinizing hormone

## Discussion

The quantitative and objective measurements of progesterone levels are essential for assessing the reproductive status in bitch, especially optimal breeding time, and predicting or monitoring parturition. RIA has long been used as the gold standard method to measure the value of progesterone in bitches [18-21]; however, in 2014, the CLIA method was accepted and became popular for progesterone measurement [22-24]. CMIA is a modified and advanced form of CLIA and used by veterinary reference laboratories in Thailand. Limitations of both CLIA and CMIA were that they take several hours to several days for processing, depending on the location of the laboratory. However, in-house progesterone measurement is trending as a tool for veterinarian practitioners because it is simple, convenient, and rapid.

The present study showed a good correlation of progesterone measurement in the same serum sample for both RFICA and CMIA assays. These results indicated that in-house progesterone measurement using RFICA demonstrated a high correlation to the veterinary reference laboratory using CMIA. Five critical measurement times during estrous in bitches showed statistically significant differences, except at the fertilizable period, which showed a non-significant difference, indicating no difference between both measurement assays. The precision of progesterone measurement must be calculated using the equation of linear regression. However, in the fertilizable period, there is no need to calculate the precision of progesterone measurement using RFICA because at this level, there was no difference using both measurement assays.

Based on these results, it was concluded that using RFICA instead of CMIA to determine the concentration of progesterone in bitch sera required a change in the target concentrations associated with critical events during estrus as well as a change in the interpretation of the temporal relationship between reaching the respective target concentration with both assays. Therefore, RFICA was made the reference or guideline for optimal breeding time interpretation (Table-2).

**Table-2:** Reference or guideline for progesterone interpretation using RFICA in heat or apparent reproductively quiescent bitches.

Progesterone (ng/mL)	Likely events	Action
<2	Anestrus, proestrus, and pre-LH surge	Retest in 2-3 days
2-3	LH surge	Retest in 2 days to confirm continued rise in progesterone. (aim for breeding 4-7 days)
3-4	Post-LH surge and pre-ovulation	Retest in 1-2 days to confirm continued rise in progesterone. (aim for breeding 3-5 days)
4-10	At or near ovulation	Retest in 1 day to confirm continued rise in progesterone. (aim for breeding 2-4 days)
10-40	In fertilizable period	Aim for breeding on this day and for another 2-3 days hereafter.

RFICA: Rapid fluorescence immunochromatography assay, LH=Luteinizing hormone

## Conclusion

The present study demonstrated that it is ­presumably acceptable to use the RFICA and CMIA methods interchangeably for quality progesterone measurements in serum samples from bitches. However, when considering the use of the RFICA method, it is advisable to interpret the results carefullyand follow the interpretation guidelines as per Table-2. Finally, RFICA in the present study provides a reliable and convenient option for veterinarian practitioners to measure canine progesterone in-house.

## Authors’ Contributions

NK, DP, and SR carried out the main research works and analyzed the main data in the experiments. SR supervised the laboratory work and approved the final version of the manuscript. All authors have read and approved the final manuscript.
